# Relative Contributions of Solubility and Mobility to the Stability of Amorphous Solid Dispersions of Poorly Soluble Drugs: A Molecular Dynamics Simulation Study

**DOI:** 10.3390/pharmaceutics10030101

**Published:** 2018-07-21

**Authors:** Michael Brunsteiner, Johannes Khinast, Amrit Paudel

**Affiliations:** 1 Research Center Pharmaceutical Engineering Gmbh, 8010 Graz, Austria; michael.brunsteiner@rcpe.at (M.B.); khinast@tugraz.at (J.K.); 2 Institute of Process and Particle Engineering, Graz University of Technology, 8010 Graz, Austria

**Keywords:** molecular dynamics simulation, amorphous, physical stability, hydrogen-bond, molecular mobility, mixing energy, molecular interactions

## Abstract

Amorphous solid dispersions are considered a promising formulation strategy for the oral delivery of poorly soluble drugs. The limiting factor for the applicability of this approach is the physical (in)stability of the amorphous phase in solid samples. Minimizing the risk of reduced shelf life for a new drug by establishing a suitable excipient/polymer-type from first principles would be desirable to accelerate formulation development. Here, we perform Molecular Dynamics simulations to determine properties of blends of eight different polymer–small molecule drug combinations for which stability data are available from a consistent set of literature data. We calculate thermodynamic factors (mixing energies) as well as mobilities (diffusion rates and roto-vibrational fluctuations). We find that either of the two factors, mobility and energetics, can determine the relative stability of the amorphous form for a given drug. Which factor is rate limiting depends on physico-chemical properties of the drug and the excipients/polymers. The methods outlined here can be readily employed for an in silico pre-screening of different excipients for a given drug to establish a qualitative ranking of the expected relative stabilities, thereby accelerating and streamlining formulation development.

## 1. Introduction

A substantial percentage of small molecule drugs in development pipelines are expected to have poor aqueous solubilities and thus inadequate oral bioavailabilities [1]. As the preferred type of drug formulation is usually the solid oral dosage form, low solubility can be a serious issue for the developability of a new active pharmaceutical ingredient (API). A potential remedy is the formulation of drugs as amorphous solids, a strategy that can improve aqueous solubilities due to the higher free enthalpy of API molecules in the amorphous compared to the crystalline state. However, at ambient conditions, small molecule drugs are usually more stable in their crystalline compared to the amorphous state. Such amorphous solids are meta-stable at best, and their conversion into crystalline solids, and the concomitant reduction in solubility, is only a matter of time. Consequently, this strategy has been successfully applied in only a small number of cases to date as ensuring the required physical (long term) stability of such formulations can be difficult [2].

A popular strategy towards improving the physical stability of amorphous drugs has been the preparation of amorphous solid dispersions (ASD), i.e., their co-formulation with intrinsically amorphous excipients, usually polymers such as poly-vinyl-pyrrolidone (PVP) or hydroxypropyl methylcellulose (HPMC) [2,3,4,5]. Due to the large chemical variety of drug compounds, their miscibility with with various polymer types can vary widely, and thus for each new API its compatibility with different polymers needs to be established at the onset of formulation development. Several different experimental and theoretical methods have been proposed and used for this purpose. On the experimental side, this includes thermal analysis, via melting point depression (DSC), thermo-rheological methods, recrystallization, or dissolution end point methods [6,7,8]. If a liquid low molecular weight analogue of the polymer is available, relative drug solubilities can also be estimated by measuring solubilities in this analogue [9]. This, however, requires that such an analogue exists, which is not necessarily the case for all commonly used polymers, and it also cannot account for the effect of finite polymer chain lengths and their impact on kinetic stabilities [10,11,12]. Moreover, the latter method assumes the activity coefficient of a drug molecule in a small molecule analogue to be comparable to that in polymer counterpart at a given concentration. Common to most of these methods is that room temperature drug solubilities in polymers are not measured directly, and the interpretation of experimental results is based on various assumptions and models which might apply in a given case or not. In a recent review and comparison of these methods, Knopp et al. concluded that relative solubilities obtained with different methods do not agree in all cases, and the optimal choice of experimental method for a determination of solubilities depends on the thermal properties of drug and polymer [11].

Next to the experimental effort, the techniques mentioned above require a substantial amount of API, a commodity that can not be taken for granted at the early pre-formulation stage. Thus, a pre-ranking of various polymers with respect to the expected stability of the ASD with a given API would be beneficial as a means to streamline and accelerate formulation development. For this purpose, several theoretical methods have been proposed. A comparatively simple approach is a statistical analysis of the correlation between various molecular descriptors of the API and the stability of an ASD with a given polymer. Moore at al developed such a model for PVP using the descriptors based on EDRAGON [13] and stability data of 12 API molecules. They identify one descriptor, called R3m index, showing an excellent correlation with stability [14]. However, the authors stated: “a direct physical interpretation of the correlation between the R3m index and amorphous molecular solid dispersion potential is not readily apparent”. In addition, although they go through some effort demonstrating the statistical significance of their result, we consider it questionable whether a model based on 12 data points, and choosing one out of several thousand different descriptors can be expected to hold for a wide class of API molecules. A similar model based on other descriptors was proposed by Nurzynska et al., but this is only valid for pure compounds and does not take into account the effect of polymers or other excipients [15].

Another approach that has a long history, and whose physical interpretation appears to be more straight forward is the use of solubility parameters (Hansen and Hildebrandt), usually in the context of Flory–Huggins (FH) theory [16,17]. Originally developed for a description of dilute polymer solutions, more recently FH theory was embraced in formulation development as a means for the interpretation of experimental data [18,19,20,21]. However, as early as 1951, it was argued that “The lattice model, basis of the Flory–Huggins theory and equation, was at first widely accepted because it seemed to be in agreement with the available data [...] with only one adjustable parameter, the Huggins Φ constant. With more recent work [...] serious discrepancies in the theory have become evident. More thorough weighing of the theory at the outset [...] might have led to the expectation that it would fail” [22]. Strikingly, now, more than sixty years later, this assessment seems to have been largely forgotten and ignored. Specific and directional intermolecular interactions of varying strength, in particular hydrogen (H) bonding exist in most drug–polymer systems [23,24,25]. Quantitative values of the strength of such specific interactions and the degree to which they influence thermodynamic and kinetic properties remain unaccounted for in these models, resulting in poor miscibility predictions for interacting composites [10]. A conceptually different approach is the perturbed-chain statistical associating fluid theory [26]. It was applied to estimate the stability of a number of amorphous APIs [27], but the effect of excipients/polymers has not been accounted for. In addition, the method requires empirical parameters that are not always readily available for new compounds. For example, fluid-state properties of high polymers are quite challenging to measure and also the impact of chirality/tacticity on the directional interactions such as H-bonding are hard to account for.

An alternative theoretical method for an estimation of relative stabilities of an API in various polymer types are models based on atomic scale molecular simulations. In principle, such models could provide both a ranking of different polymer types with respect to the stability of the ASD with a given API and insights into the physical mechanism that provides this stability. Gupta et al. performed MD simulations of blends of Celecoxib and PVP [28]. They reported the observed interactions between specific API and polymer functional groups and confirmed these findings using spectroscopic methods. Anderson and co-workers performed molecular dynamics (MD) simulations of indomethacin in a PVP matrix. They identified the changes in H-bonding patterns upon mixing and used the calculated energies to parameterize a FH interaction parameter [29]. However, in neither of the accounts mentioned above, were attempts made to extend the method to cover more than a single API–polymer combination to investigate its accuracy in the prediction of relative stabilities. Jha et al. used MD simulations to study molecular interactions between a model drug and two different cellulose based polymers in aqueous solutions at different concentrations. They investigated structural features and give some general recommendations regarding preferential substituents on the polymers, but no comparison with experimental stabilities was included [30]. In a somewhat different approach, Maniruzzaman calculated interaction energies at the ab initio level between dimers of several polymers and different APIs performing in silico energy minimizations of small drug–polymer complexes. However, no clear correlation between miscibilities or stabilities and the calculated energies was apparent [31]. Gupta et al. determined the relative stabilities of ASDs of indomethacin, with polyethylene oxide, glucose, and sucrose by calculating solubility parameters via MD simulation of the pure API and excipients [32]. No simulations of blends were included, and the resulting model is expected to suffer from the same limitations as the above mentioned solubility parameters/FH based methods, not accounting for specific intermolecular polymer–API interactions.

**Figure 1:**
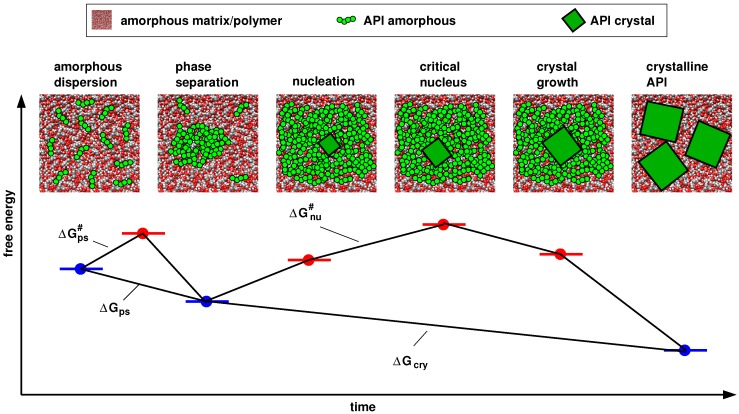
Schematic presentation of the free energy landscape of an amorphous solid dispersion undergoing molecular relaxation, phase separation, nucleation and crystal growth.

The examples mentioned above could certainly provide valuable insights in specific cases, but they are limited in scope, and so far comprehensive and comparative studies demonstrating the general usefulness of this approach are not available. In addition, most molecular modeling studies towards the stability of ASDs published so far concentrate on the thermodynamic aspect, i.e., they consider equilibrium properties, mixing enthalpies and H-bonding. However, as the solubilities of drugs in polymers are often lower than the required drug loads, we are facing non-equilibrium systems with time-dependent properties, and a stability that is governed by kinetics and relaxation processes [33,34,35] (Figure 1). The relative stabilities of different amorphous systems or glasses have been associated with both α-relaxation (translational diffusion) [36] and higher order mobilities (Johari–Goldstein and β-relaxation) [35]. One example including amorphous drugs is given in a recent publication by Knapil et al. Using various spectroscopic methods and DSC, the authors demonstrated for a co-amorphous system of two API molecules at different molar ratios that stability of the amorphous state clearly correlates with molecular mobilities [37]. To our knowledge, a comparative study using atomic scale molecular simulation to investigate the impact of both effects, thermodynamics and kinetics, on the stability of a range of different API–polymer combinations has not been available to date. Even most experimental accounts reported so far concentrate on either the thermodynamic solubility of API in polymer or on the molecular mobility.

**Figure 2:**
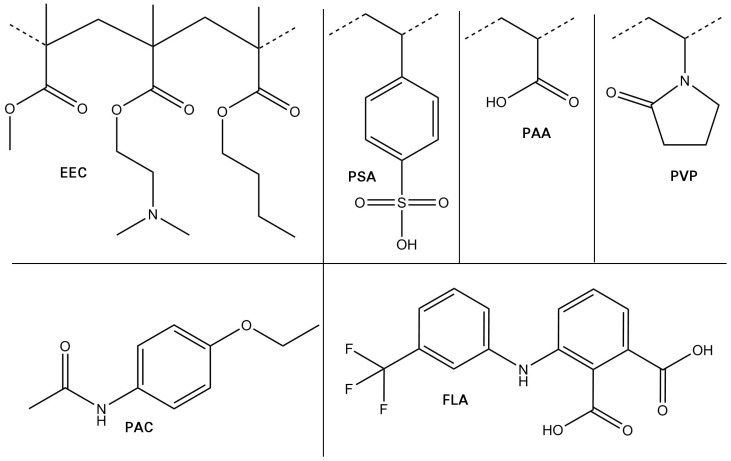
Compounds used in this study: (**top**) Polymers: eudragit (EEC), polystyrene sulfonic acid (PSA), poly acrylic acid (PAA), and poly vinylpyrrolidone (PVP); and (**bottom**) APIs: phenacetin (PAC) and flufenamic acid (FLA).

While being related, the molecular basis of thermodynamic and kinetic contributions to the physical stability of ASDs have not been reported. In the present contribution, we aim to develop and deploy MD simulations to derive thermodynamic (energetic) and kinetic (mobility) descriptors for diverse ASDs and compare the outcome with the reported experimental study. To this end, we report first results obtained by performing and analyzing extensive MD simulations of two different API molecules, namely flufenamic acid (FLA) and phenacetin (PAC), each blended at two different compositions in ASDs with Eudragit E100 (EEC), polyacrylic acid (PAA), poly (styrene sulfonic acid) (PSA) and PVP (Figure 2). Though experimental solubility values vary, both APIs can definitely be considered poorly soluble (S ⪅ 1.0 × 10${}^{-3}$ mol/L) [38]. For each API–polymer combination, we determined mixing energies, variations of H-bonding, and API mobilities in the blend. Here, we compare our results to experimental stabilities from literature data, and discuss the relative impact of both factors, thermodynamics and kinetics, on stabilities. Finally, we interpret our findings on the basis of the API molecules’ molecular structures and physicochemical properties.

## 2. Methods

### 2.1. Force Field

A crucial ingredient of classical molecular simulation are the parameters of the semi-empirical equations that are used to calculate energies and forces for a given structure, usually referred to as the force field. We used the General Amber Force Field (GAFF), which has been shown to reproduce a range of properties for a wide spectrum of organic molecules [39]. Ambertools [40], acpype [41], and the amb2gmx perl script [42] were used to identify atomtypes, assign bonded and Lennard–Jones parameters, and convert Amber to Gromacs topology files. Partial charges for each atom where determined from electrostatic potential derived charges in a set of ab initio calculations at the DFT-B3LYP level of theory using the cc-pVTZ basis set and a solvation correction with a dielectric constant of 4 [43]. For these calculations, we used the RED online server [44] as well as Gamess-US [45] on local workstations. For the conversion of the resulting charge density distributions to partial charges, we used the RESP algorithm implemented in the Ambertools software, version 16. [40]. For the polymers, the ab initio calculations were performed using trimers, in each case four different conformations. Considering that all simulated samples are in the solid state without water, we decided to model all molecules, APIs and polymers in their neutral state with zero net-charge.

For the two API molecules considered, the resulting force fields were tested by performing short 1-ns MD simulations of the crystals at ambient conditions using Gromacs [46]. Initial structures were generated by replicating the unit cell of the the crystal structures of the most stable polymorph of each API to obtain supercells of sufficient size, i.e., with a minimum extension of 4 nm in each dimension. MD simulations of these systems at ambient conditions were performed and the root mean square deviation between the averages structures from the simulation and the experimental crystal structures was calculated. The resulting numbers converged around 1.2 Å for FLA, and 0.6 Å for PAC. These numbers as well as visual inspection of the trajectories confirmed that the force field can faithfully reproduce at least structural features of the API compounds studied here.

### 2.2. Molecular Dynamics Simulations

To reproduce the effects discussed in the publication of the experimental data used here as faithfully as possible, and to generate results for different blends that are as comparable as possible, we attempted to produce blends of polymers and APIs that: (i) have approximately the same polymer–API molar or weight ratios as used in the experimental study; and (ii) have comparable numbers for the total weights and volumes. Thus, we produced 16 different systems (2 APIs × 4 polymers × 2 concentrations). The concentrations we chose to use correspond to 25 and 40 weight percent API. The polymers were modeled as atactic chains consisting of 40 monomer units. In the case of eudragit EEC, which is a co-polymer, the ratio of the monomer units was used as specified by the manufacturer of this polymer (dimethylaminoethyl methacrylate, butyl methacrylate, and methyl methacrylate with a ratio of 2:1:1) and the order of monomer types was chosen randomly. The total system size corresponds to a mass of about 80 kD for the polymer plus the corresponding mass (25 or 40 wt %) for the API. Details for the molecular contents of the blends and the pure samples are provided in Table 1. All initial structures were generated using in-house scripts by placing polymers, initially extended chains, and API molecules, both with a random orientation, approximately evenly distributed in a box that was large enough to exclude any major overlaps between neighboring molecules. For each polymer–API combination and concentration, four such structures were generated by varying the orientation and initial velocities of all molecules and atoms, respectively. These 64 systems (2 APIs × 4 polymers × 2 concentrations × 4 copies with different structures) were subjected to a short energy minimization run, followed by several cycles of compression, heating, and quenching (1–1000 bar and 300–1000 K) to produce ASDs with roughly evenly distributed partially entangled polymers and APIs at realistic densities. The procedure covered about 20 ns simulation time for each system. This was followed by an equilibration phase, an MD simulation at ambient conditions for another 100 ns, and production runs at the same conditions of varying length (0.2–1.4 μs). For simulations of samples of pure polymer and pure API, initial structures were generated in a similar manner.

All MD simulations were performed using GROMACS (versions 4.6.5, 5.0.4., and 5.1.2) [46]. For integration of the equations of motion a velocity verlet algorithm with a time step of two femtoseconds was used. Temperature and pressure were controlled using the Nose–Hoover thermostat [47], and Berendsen barostat [48], respectively. A cut-off radius of 9 Å was used for Lennard–Jones and electrostatic interactions. Electrostatic long range interactions were calculated using a Smooth Particle Mesh Ewald (PME) algorithm [49]. For dispersion interactions beyond the cut-off range, a correction factor was included. All bonds including hydrogen atoms were constrained using the LINCS algorithm [50]. Snapshots of the system were saved at intervals of two picoseconds.

### 2.3. Analysis

The MD trajectories were analyzed to determine energies, H-bonding, and mobilities using various tools and algorithms that are part of the GROMACS distribution as well as a number of in-house scripts. Unless mentioned explicitly, all numbers reported below are averages from four independent simulations with different initial structures and initial particle velocities. Error bars were determined as standard deviations calculated from these four averages. Two specific aspects of the analysis should be mentioned in more detail:

*Interaction energies:* Due to the nature of the PME algorithm, the Coulomb contribution to inter-molecular interaction energies (E${}_{Coul}$) for different components of a mixture cannot be directly calculated from a single analysis of the trajectory. For this purpose, the energies needed to be re-calculated threefold: (1) for the original system; (2) for the original system with all charges on the interesting molecule set to zero; and (3) for the original system with all charges but those on the interesting molecule set to zero. This threefold re-calculation needs to be performed for the entire trajectory and for each molecule in turn to obtain average E${}_{Coul}$ values that contain only inter- but no intra-molecular interactions, and the correct contribution of electrostatic long range interactions.

*Contributions to mobility:* Calculation of the translational component of molecular mobilities is straight forward, using the average squared distances of molecules centers of mass as function of time. To obtain average values for the mobility involving rotational and vibrational degrees of freedom of the API molecules, we proceeded as follows: the trajectories were split into parts, one for each API molecule. Subsequently, each of these sub-trajectories of a single API molecule was processed so that the center of mass of the API molecule was moved to origin, keeping its conformation and orientation intact. The resulting trajectories were then analyzed using the GROMACS rmsf tool [46] to calculate the average root mean square fluctuation of each atom in the molecule around its individual average position. the resulting values for each non-hydrogen atom were averaged for all atoms in all molecules to obtain a number referred to as RMSF below. We used this number as a measure for the lump sum of the higher order (β, γ, etc. relaxation) mobility of the API.

## 3. Results

### 3.1. Choice of Model Systems

One issue hampering progress in the development of improved models for the prediction of ASD stabilities is the scarcity of comparable experimental data. Most of the existing experimental accounts only discuss results for a single, or a small number of API–polymer combinations, and a comparison of numbers from different studies, obtained with different experimental procedures is obviously rather difficult. Here, a notable exception is the data published by Van Eerdenbrugh and Taylor who determined and compared the stabilities for good number of different API–polymer combinations, using in all cases the same experimental protocol [51]. The authors attempted to explain their data on the basis of molecular properties, in particular on the presence and combination of H-bond donors and acceptors of a given strength in drug and polymer, respectively. The data used here for comparison with results from molecular modeling of API–polymer blends are so-called amorphicity indices (AI), that were determined by Eerdenbrugh and Taylor for combinations of eight different API molecules and seven different polymers. AI values are dimensionless numbers ranging from 0 to 100, and a measure for the relative amorphous content observed in an ASD after a given storage time at room temperature. The higher the number the more stable a particular choice of API–polymer combination is expected to be. In practice AI values were determined for samples prepared by spin coating by visual inspection under polarized light microscopy and based on the degree of birefringence observed. For more details, we refer to the original publication [51].

The computational effort of the simulations reported here is considerable. Therefore, we chose to use only a subset of the data provided in the work by Van Eerdenbrugh and Taylor [51]. First, we discarded all combinations with HPMC and HPMCAS as polymers, since, in most cases, results with these polymers lie intermediate in between some of the other polymers, and trends are less pronounced. Moreover, polysaccharides such as cellulosic polymers can be difficult to model reliably with empirical model potentials, compared to non-sugar organic molecules [52,53]. We also discarded data from PVPVA based ASDs since here the results were qualitatively identical to those obtained with PVP, leaving four polymers: Eudragit E100 (EEC), poly acrylic acid (PAA), poly sulfonic acid (PSA), and PVP. We then visualized the data as shown in Figure S1 to identify groups of API molecules representing the same stability trends and similar chemistries. Data for bifonazole was discarded as this API showed essentially the same AI value for each of the four polymer types. Each of the remaining APIs comprises a comparatively rigid aromatic ring system with a varying number of substitutes including amide, carboxylic acid and and halogen groups. They can be divided into two groups, one with molecules that feature a strong donor (chlorzoxazone, flufenamic acid, flurbiprofen, and chlorpropamide), and a second with weak or intermediate donors (lidocaine, benzamide, and phenacetin). The molecules within each group display similar trends with respect to their relative stabilities with the four polymers. Molecules of the first group are more stable with PVP and EEC, than with PAA and PSA; molecules of second group show poor stability with EEC, and good stabilities with each of the three other polymers. From each group, we chose the molecule for which the most pronounced differences in stabilities were observed, for the first group flufenamic acid (FLA), and for the second phenacetin (PAC).

### 3.2. Convergence

The systems considered here are essentially glasses, i.e., non-equilibrium systems. Thus, they are subject to aging, a process whose completion, even for the small system sizes considered here, can take much longer than the comparatively short time scales that are achievable with atomic scale molecular simulation. Unless the solubility of an API in a given polymer is equal or above the concentration in the initial structure mixing energies are therefore time-dependent and essentially ill-defined. The resulting enthalpy and density relaxation has been observed before for similar systems [54]. However, if we are only interested in relative energies, i.e., a qualitative ranking for systems of a given API combined with various polymers, we can assume that this ranking will not change after an initial period. To improve the probability of being in this regime where relative energies stay reasonably constant, we performed rather long MD simulations runs that compare favorably to previously published accounts.

For each of the systems reported here numbers were obtained as averages and standard deviations of four MD simulations with different starting geometries and initial velocities. Each single simulation of pure compounds was extended to cover 200 ns (APIs) and 400 ns (polymers), respectively. The time development of energies and volumes is shown in Figures S2 and S4 in the SI. Not surprisingly we find that even after these comparatively long simulation times it is unclear whether full convergence is achieved. However, a comparison of the time developments in a single diagram (Figures S3 and S5) suggests that the relative numbers show reasonably good convergence, Simulations of different polymer–API blends with a weight ratio of 25 wt % API were extended to cover 400 ns. Again visual inspection of the time developments of the individual simulations, (Figures S6 and S8) and their comparison in a single diagram (Figures S7 and S9) suggest reasonable convergence of the calculated relative numbers. The results obtained at a weight ratio of 40 wt % API were extended to cover one microsecond. Here, convergence appears to be better than it is at the lower API concentrations. (Figures S10–S13) Using averages from the time intervals 150–200 ns (APIs), 200–400 ns (polymers and blends at 25 wt %), and 600–1000 ns (blends at 40 wt %), we expect to obtain reproducible numbers at least for relative energies, i.e., trends for a given API combined with different polymers.

As opposed to mixing energies the mobility, here calculated as diffusion coefficients for the API molecules in the different polymer matrices, should show better reproducibility and convergence. However, the low mobility of API molecules in this type of system combined with the overall small system sizes renders achieving converged results difficult. Better convergence is observed for the simulations with API concentrations of 40 wt % (one microsecond simulation time) compared to 25 wt % (400 ns simulation time), but even here the final numbers for the calculated API diffusion coefficients are within each others error-bars for FLA (Figure S14). For PAC, we extended the simulations to each cover 1.4 μs (Figure S15) and here, in comparison, we observe significant differences, which are discussed below.

### 3.3. Energy Terms and Trends

The estimated relative polymer–API mixing energies comprise one of the two major descriptors of molecular miscibility, and thereby stability, considered here. Due to the nature of classical force fields, a number of different energies can be calculated from MD simulations. The energy terms that are parameterized by the force field used here are typical for classical model potentials and given in Equation (1).

(1) $$E_{tot}=E_{bond}+E_{angle}+E_{dih}+E_{LJ}+E_{Coul}$$

They include so-called bonded interactions: bond (E${}_{bond}$), angle (E${}_{angle}$), regular and improper dihedral terms, E${}_{dih}$, as well as non-bonded interactions: Lennard–Jones (i.e., Van der Waals, (VdW) E${}_{LJ}$, and Coulomb (E${}_{Coul}$) energies. The two latter can be subdivided into inter- and intra-molecular contributions. A special case are the so-called 1–4-interactions, which are, usually scaled, VdW and Coulomb interactions between atoms in a given molecule that are separated by three bonds. The best choice for a calculation of mixing energies to be compared with experimental stabilities appears to be E${}_{tot}$, the sum of all these energies. However, if we consider the way in which classical force fields are parameterized, we will find that some of these contributions might be more specific and/or more accurate than others. In particular the Lennard–Jones (VdW) parameters are often the result of fitting procedures with little physical basis. In the present case, i.e., for the GAFF force field, they were transfered unmodified from the original Amber peptide parameters based on chemical similarities. Whether relative dispersion energies of different molecular combinations can be reproduced even semi-quantitatively is unclear. Bond, angle, dihedral, and in particular 1–4 interactions are generally the result of fitting procedures aimed at reproducing experimental structures rather than energies. Thus, concentrating on inter-molecular interactions only might provide more reliable results than inclusion of all terms. In addition, these energies are expected to represent experimentally measurable sublimation enthalpies (cohesive energies).

Another open question regarding the quantities to compare experimental data with is the normalization of energy terms, and the choice of reference state. For a sample of a pure compound, normalization appears to be trivial. The total calculated energy of a given sample is simply divided by the number of molecules in the simulated sample. However, if we want to compare energies of samples with molecules of appreciably different sizes and/or energies of different mixtures this choice is less straight forward. A common remedy used here is to replace energies by energy-densities, i.e., the calculated total energy for a sample is divided by the volume of this sample. Again, if the compared samples feature appreciably different densities and/or API concentrations this might not be the optimal choice. Alternatively, and in particular when considering ASDs of drug molecules, as done here, we might want to look at energies divided by the number of drug molecules, since usually we aim at a high drug load per sample. As for the reference state we can choose comparing the total energies (or energy densities) of different blends (E) or the energy differences (ΔE) between the mixture and a (sum of) reference state(s). This reference state can be the energy of a given molecule in the gas phase, in the amorphous solid, or in the crystalline solid—or the weighted sum of such energies in pure samples if we compare mixtures.

**Figure 3:**
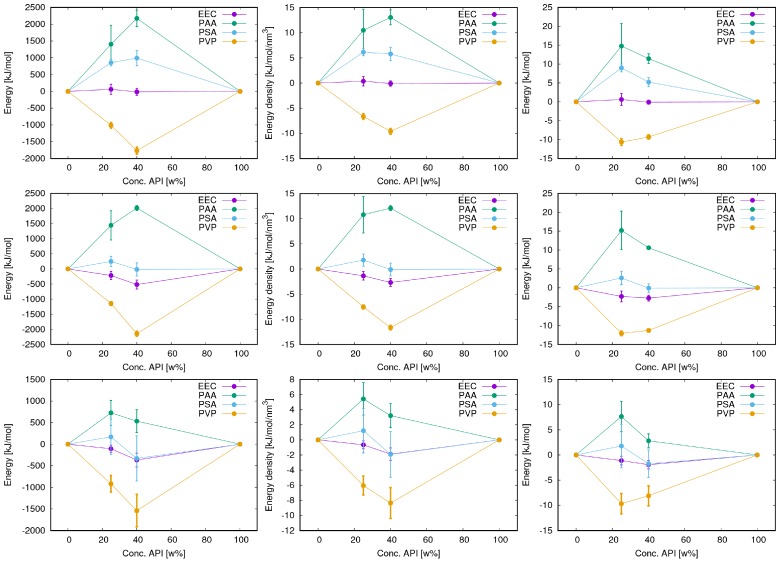
Variation of Energy terms with API concentration for FLA: (**left**) total energy difference; (**center**) normalized by volume; and (**right**) normalized by number of API molecules in blend. From **top** to **bottom**: E${}_{Coul}$, E${}_{nb}$, and E${}_{tot}$.

For none of the questions outlined above there appears to be an un-ambiguous answer. Here, we calculated, and compared three types of energies: ΔE${}_{tot}$, ΔE${}_{nb}$, and ΔE${}_{Coul}$. E${}_{nb}$ (nb stands for non-bonded) is the sum of all VdW and Coulomb interactions, including intra-molecular VdW and Coulomb contributions, excluding 1–4 interactions. As reference state, we chose, in all cases, the sum of the energies of the same amount of molecules (API and polymers) in the pure amorphous phases. Thus, the resulting energy difference corresponds to ΔE${}_{ps}$ in Figure 1. Additionally, we normalized each energy difference by the sample volume, or the number of API molecules. Un-normalized values are also provided. Results for FLA are shown in Figure 3. We find that, in all cases, the observed trends for a given API, i.e., the relative energies in blends with different polymer types, are identical, irrespective of the energy term or the type of normalization. This is probably a consequence of the fact that we chose to make the various blends, regarding their composition, as comparable as possible (see Section 2.2), and that the total densities of all samples are fairly similar. It also suggests that the relative electrostatic interactions dominate the differences between different blends as this energy contribution is part of all three energy terms shown in Figure 3. This was to be expected as electrostatic interactions usually represent the largest intermolecular energy contribution in such systems that feature a substantial amount of H-bonds. The combination of different structures and charge distributions also results in large variations of this term and will, therefore, dominate the relative compatibilities of different API–polymer combinations. The equivalent type of diagrams for PAC (not shown) confirm this conclusion.

Individual numbers for the energies calculated at the two API concentrations considered here (25 and 40 wt %) differ. However, the trends (relative numbers) obtained with the four polymers for a given API are the same and do not vary with API concentration. Therefore, in the following, we only discuss results obtained for one concentration, where we chose the 40 wt % samples since here usually the statistics, i.e., the precision of the results is better.

**Figure 4:**
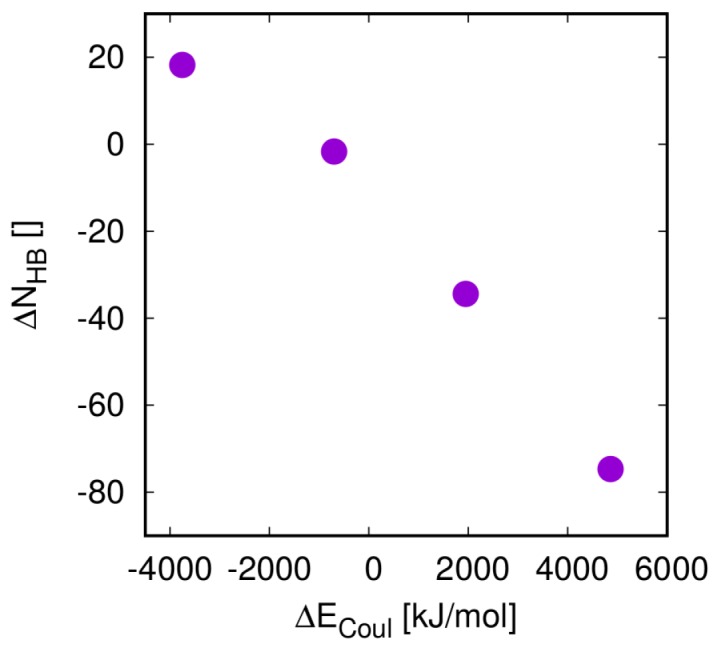
Correlation between the change in Coulomb interaction energy ΔE${}_{Coul}$ and change in the number of H-bonds (ΔN${}_{HB}$) in blends of 40 wt % FLA with PVP, EEC, PSA, and PAA (from higher to lower values of ΔN${}_{HB}$). Numbers are the differences between the quantities in the mixtures and of the equivalent numbers of molecules in the pure phases.

As a substitute for energies, a structural parameter, the change in the number of H-bonds upon mixing (ΔN${}_{HB}$) is sometimes employed as a criterion for solubility [29,55]. For all pure samples and blends, we calculated this number as outlined in Section 2.3. ΔN${}_{HB}$ shows an excellent inverse correlation with ΔE${}_{Coul}$. For FLA at 40 wt % and the four polymers considered here this correlation is shown in Figure 4. For the remaining systems considered here, this correlation is not shown, but is is in all cases good, with a Pearson correlation, r>0.8, and in most cases excellent with 0.9<r<1.0.

In the systems considered here, hydrogen bonds provide by far the largest contribution to the overall Coulomb energies, thus the correlation between ΔE${}_{Coul}$ and ΔN${}_{HB}$ is no surprise, and we expect this relation to hold for most systems with comparable chemistry. The good correlation between these terms suggests that calculation of only one of the two terms is required to capture the corresponding physics. In the following, we therefore only report ΔE${}_{Coul}$. Relations between ΔN${}_{HB}$ and amorphous stabilities are not shown as they are in all cases, at least qualitatively, equivalent to those of ΔE${}_{Coul}$.

### 3.4. Flufenamic Acid

**Figure 5:**
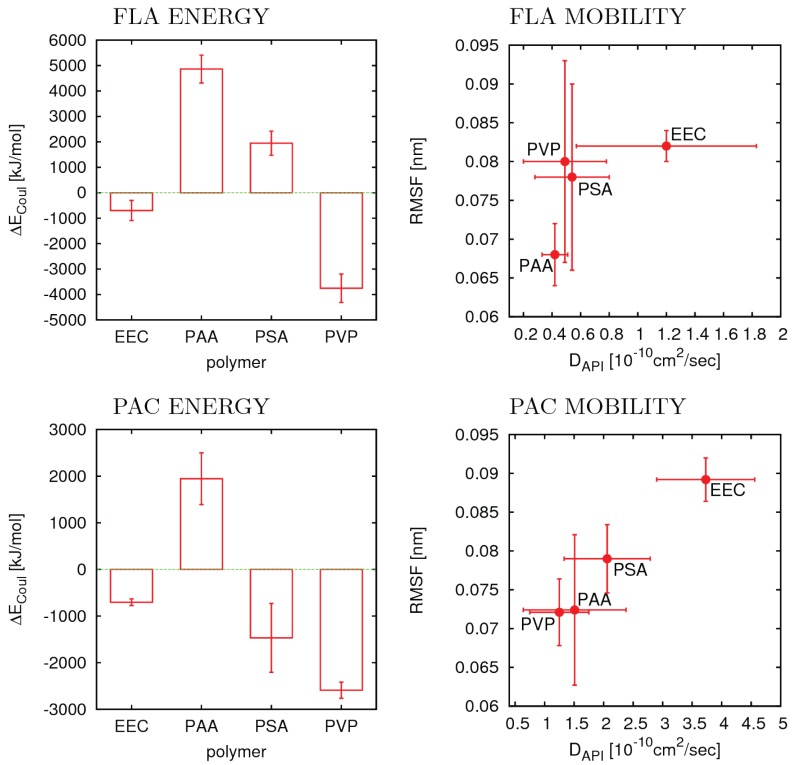
Results from MD simulations of API (40 wt %) blends in four different polymer matrices: FLA (**top**); and PAC (**bottom**). The two diagrams on the left show the changes of the Coulomb interaction energies upon mixing. The diagrams on the right show API translational diffusion coefficient and roto-vibrational mobility (RMSF). Each point is labeled with the corresponding polymer type. The error bars are standard deviations calculated from four replicates.

Results for FLA, ΔE${}_{Coul}$ and for the API mobilities in the polymer matrices, are shown in top of Figure 5 and Table 2. We found that ΔE${}_{Coul}$ of FLA in PSA and PAA is positive (unfavorable) while in EEC and PVP negative (favorable) contributions to the mixing energies are obtained. This is in agreement with the experimental observation that PSA and PAA provide ASDs with comparatively good stabilities (AI = 100) while mixed with the two former polymers the API shows pronounced crystallization tendency (AI ≤ 0.13). ΔN${}_{HB}$, the change in the number of H-bonds (not shown) follows the same trend. For the mobility of the API in the polymer matrix, two different estimates are provided: the translational diffusion coefficient of the API (D), and the average root mean square fluctuation (RMSF) of all atoms in the API molecules, calculated as described in Section 2.3. The latter we use as a coarse measure for the sum of mobility contributions of higher order or local/secondary molecular motions (roto-vibrational degrees of freedom and β, γ, etc. relaxation). No correlation with experimental stabilities can be observed simply due to the fact that values are so similar that, in most cases, the error bars overlap. The vague trend suggesting higher mobility, and thus poorer stability, for EEC does not agree with experimental data. The results in Figure 5 suggest that the relative stabilities of FLA in the four polymer types considered here are predominantly determined by thermodynamics (relative mixing energies) rather than kinetics. This is basically in line with the interpretation in the original publication of the experimental results, which assigns the good stability of FLA in EEC and PVP to the strong H-bonds that can be formed between the API and the two polymers, or actually the larger energy gain through mixing a strong H-bond donor with a polymer that has only acceptors and no donors, and therefore cannot form any H-bonds in the pure phase.

### 3.5. Phenacetin

For PAC results for ΔE${}_{Coul}$ and for the mobility of the API in the polymer matrices are shown on the bottom of Figure 5 and in Table 2. As opposed to the FLA cases the mixing energy or its electrostatic contribution can not explain the experimental trend observed for the relative stabilities of the four API polymer blends. The calculated energies suggest that PAA shows the poorest, and PVP the best performance in terms of miscibility with PAC. EEC and PSA show intermediate performance. The experimental stability data, however, shows that three of the four polymers, PAA, PSA, and PVP provide relatively similar and good stabilities when blended with PAC. Only EEC has a significantly poorer performance compared to the others. This suggests that thermodynamics plays no, or a minor, role in the relative stabilities of PAC blended with the four polymer types. If this is true, then the kinetic stabilities, or the relative molecular mobilities of the API molecules must be the rate limiting factor. Indeed, if we consider the mobilities of PAC in the four polymer matrices as shown in Figure 5, we find that numbers for PAA, PSA and PVP basically lie within each others error-bars, and only in EEC PAC shows a significantly higher mobility compared to the others, which qualitatively agrees with the available experimental data.

## 4. Discussion

### 4.1. Thermodynamics vs. Kinetics

**Figure 6:**
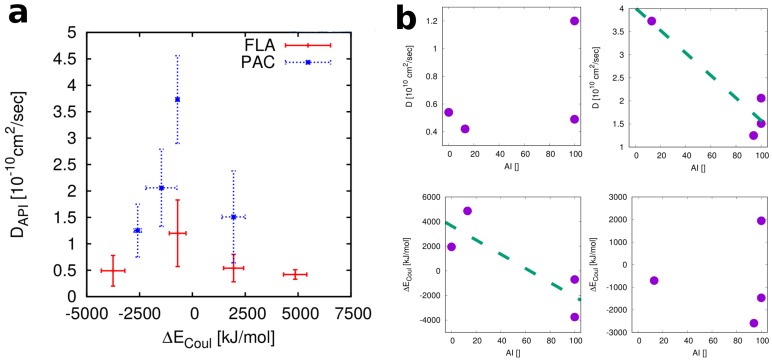
(**a**) Comparison of the ranges of ΔE${}_{Coul}$ and diffusion coefficients for FLA vs. PAC. The polymers are in the order of increasing ΔE${}_{Coul}$: PVP, EEC, PSA, PAA (for FLA), and PVP, PSA, EEC, PAA (for PAC). (**b**) Correlations between calculated descriptors (top: API Diffusion coefficient, bottom: Coulomb contribution to the mixing energy) and amorphous stabilities (AI values for 40% drug-load) for FLA (**left column**) and PAC (**right column**). The dashed green lines are included as guide for the eye.

Our results suggest that thermodynamic factors are rate limiting for the relative stabilities of FLA in the four polymers considered here, while those of PAC are determined by kinetic factors. This conclusion is also supported by our calculations if the numbers are plotted in a different way, as done in Figure 6a where the four ΔE${}_{Coul}$ and D values are plotted for each of the two APIs in a single diagram. We find that for FLA the energies cover a range of about 8620 kJ/mol, while for PAC the corresponding range is nearly half (4535 kJ/mol). For the mobilities, on the other hand side, we see the opposite relation: PAC in the four polymers covers a range of ΔD = 2.5 × 10${}^{-10}$ cm${}^{2}$/s while FLA only varies by ΔD = 0.8 × 10${}^{-10}$ cm${}^{2}$/s. Thus, for FLA, whose stability correlates with mixing energies, these energies show a larger variation than for the other API. For PAC, whose stability correlates with API mobility, these mobilities show a larger variation than for the other API. Generally, our calculations suggest that, irrespective of the polymer, the mobility of PAC is higher than that of FLA. This is in agreement with experimental numbers for the glass transition temperatures, T${}_{g}$. PAC (T${}_{g}=2{\;}^{\circ }C$) will be in the comparatively mobile and rubber-like state at RT, while FLA (T${}_{g}=17{\;}^{\circ }C$) is much closer to its glass transition. Since the glass transition is not a sharp boundary [56] FLA molecules can be expected to be considerably less mobile, at room temperature than PAC. The fact that, for both APIs, Eudragit based ASDs show the highest mobilities is in accordance with the experimental T${}_{g}$ values for the four polymers, with Eudragit having a considerably lower T${}_{g}$ than the three others. However, only for PAC this factor appears to determine the relative stabilities of ASDs with different polymers, while for FLA this effect is overridden by the relatively high solubilities of the API in Eudragit and PVP.

### 4.2. Relevant Properties of API and Excipient

Our findings do not exclude the possibility that both factors, energetics and mobility, contribute to the total stabilities of all the blends considered here, but the rate limiting factor for each API is different (Figure 6b). FLA is a compound with a carboxylic acid group. Since all the systems considered here are dry this functional group is mostly un-ionized and will act as a strong H-bond donor (as in the original experimental setup used by Van Eerdenbrugh and Taylor) [51]. Accordingly, and in line with the arguments in the publication that presented the experimental data, we can expect a good miscibility with polymers that feature H-bond acceptors. In addition to the strong API–polymer interaction the miscibility of FLA with PVP and EEC is increased by the fact that these strong interactions do not compete with any polymer–polymer interactions since neither of the two polymers has any donor functionality. PAC also has a donor functionality, but this is a secondary amide group, and thereby a much weaker donor than the carboxylic acid group of FLA. The donor group in PAC is also less flexible/accessible than the one in FLA, where the proton can tunnel from one oxygen of the carboxylic acid group to the other to optimize interaction energies (an effect that cannot be accounted for by classical molecular simulation). We hypothesize that for API–polymer combinations that allow for a very strong polymer–API interaction, preferably one that does not compete with equivalent polymer–polymer interactions (such as FLA with PVP and EEC), the equilibrium solubility of the API in a solid polymer matrix can be substantial. In these cases, the speed at which this equilibrium is reached, i.e., kinetics, is less relevant for stability of the blends. In other cases, for example PAC with the four polymer types considered here, the API at pharmaceutically relevant concentrations is generally above its solubility limit, and therefore kinetics, the speed at which equilibrium is reached, dominates the observed relative stabilities.

### 4.3. Some Technical Considerations

An important practical aspect of the interpretation of simulation results concerns the question which energy terms are the most appropriate for an estimation of the physical stability of molecular dispersions. We find that the qualitative conclusions remain un-changed whether we use energies normalized by number of API molecules, or by the volume. For FLA, the Coulomb contribution to the total change in inter-molecular interaction, however, showed a better correlation with stabilities, than the total energy, including VdW terms did. We assign this to the fact that electrostatic interactions and their variations between systems are larger than the VdW contributions, and using a simple Lennard–Jones potential the latter are neither very specific nor accurate. The most appropriate energy difference would, of course, be the difference between the solvation free energies of the API in the molecular dispersion and in pure API phase. Although tremendous progress has been made in recent years in the field of free energy calculations via molecular simulation, the calculation of solvation free energies of small organic molecules in a solid matrix below the glass transition temperature is still beyond our reach at this point [57,58]. A common remedy for this issue is to approximate relative free energy differences by relative (internal) energy differences. Our results for FLA suggest that for systems comparable to the ones studied here this is a reasonable approximation. One might argue that perhaps for PAC a better correlation between solvation energies and stabilities might have been achieved if entropic contributions had been accounted for. However, we consider this unlikely. Although details are still a matter of debate, it has been clearly shown that the entropy in molecular systems correlates with diffusion coefficients (or equivalently viscosity) [59,60]. Considering the numbers in Table 2, this would mean that for PAC in EEC the entropic contribution to the mixing energy would actually lower the energy (make it more favorable) by a larger amount than for the other polymers. This would make the correlation between stabilities (AI values) and energies even worse, suggesting that missing entropic contributions are unlikely to explain this lack of correlation.

A final technical point concerns the fact that we did not consider any cellulose based polymers. HPMC and HPMCAS probably comprise the most widely used classes of polymers in this context, and models for both polymers have in fact been developed by others [61,62]. However, it has been shown that the accuracy/reliability of classical force fields for modeling sugar based polymers is limited [53]. Our current work includes development of improved force fields for HPMC/AS. The outcome of these studies will be communicated in future publications, and we prefer to wait until then before attempting to simulate ASDs containing such polymers.

### 4.4. What Are Practical Implications?

The number of polymer–API combinations studied here is too small for providing quantitative thresholds of an API’s molecular descriptors that could be used to predict to which of the two categories (stability governed by thermodynamics or kinetics) it belongs. However, our data do suggest that both scenarios are possible. Given the fact that many drugs are similar to PAC in terms of H-bond donor and acceptor densities, further research towards establishing such values is definitely warranted.

The relevance for the above conclusions for pharmaceutical development is considerable. Most theoretical studies that use molecular simulation to study API stabilities in polymer excipients concentrate on intermolecular API–polymer interactions, in particular (relative) H-bonding propensities [19,28,29,31,63,64]. Since none of the polymers commonly used in the field has only donors and no acceptors, but there are several polymers (e.g., Eudragit and PVP) that have only acceptors but no donors, the goal of optimizing API–polymer mixing energies can most easily be achieved for APIs that include strong H-bond donor functionalities. However, the strongest donors, such as the carboxylic acid group in FLA, are acidic groups. For APIs featuring such groups, solubility issues can often be solved by their formulation as salt, rendering the application of an ASD as formulation strategy less attractive. However, in cases where polymer–API interactions are weaker, the calculation of molecular mobilities might be mandatory to obtain a correct qualitative ranking of an API’s stability in various polymer carriers. As stated above, due to the small sample size considered here, further research is required to substantiate this preliminary conclusion.

The fact that this strategy has not been adopted so far might be due to the exceptionally long simulation times required to obtain sufficiently precise values of diffusion coefficients at room temperature. Here, for example the calculation of the D values for PAC in four different polymers required MD simulations covering more than 20 microseconds for system sizes of around 20,000 atoms, taking several months on a small cluster with 16 nodes each comprising 8 cores. However, in light of the ever increasing speed of state of the art computers, and, in particular the increasing popularity of comparatively cheap GPU based architectures, this will become a minor problem in the foreseeable future [46,65,66,67].

Given the above considerations, it would be tempting to establish thresholds for the variation of calculated ΔE${}_{Coul}$ and/or diffusivity values above which a clear statement can be made about their impact on the relative ASD stabilities. However, some caution is required here since the magnitude of these values will, of course, depend not only on the API and polymers but also on simulation parameters, system size, and the employed force field. Thus, if we stated that a certain difference in diffusion coefficients or in Coulomb energies will indicate a significant difference for the ASD stabilities, this would only apply if not only the chemistry of the compounds was sufficiently similar to those used here, but also the calculations would have to be performed with the same simulation parameters, system sizes and force fields. Although in principle this could be done, in practice more reliable results will be obtained if experimental numbers for at least two polymers that, ideally provide rather different stabilities, are available to validate any conclusions drawn from calculated numbers for a given API. Notwithstanding the above, it should be possible to use calculations as outlined here to provide, for a given API, a coarse ranking of different polymers with respect to the expected stabilities of the corresponding ASDs.

## 5. Summary and Conclusions

We performed extensive MD simulations and analyzed the resulting trajectories in an attempt to improve our understanding of the mechanisms that govern the stabilities of two different APIs in ASDs with four different polymers. We believe that this study provides the most comprehensive account of this type to date. Not only did we perform simulations of a comparatively large set of polymer–API combinations, we also considered both energetics/thermodynamics and kinetics/mobility. We found that the relative stabilities of the two API molecules considered here are determined by different mechanisms. For FLA, which has very favorable inter-molecular interactions with two of the polymers, the resulting large range of mixing energies, and presumably its equilibrium solubility in at least these two polymers determine the stabilities. For PAC, only its relative mobilities in different polymer types can explain the trend observed for its stability in the four different ASDs. The importance of molecular mobility for the relaxation and stability of amorphous systems is widely appreciated, and has been thoroughly discussed in the literature. However, most, if not all, attempts using molecular simulation to explain the stability of amorphous drug formulations with polymer excipients found in the literature concentrate on specific intermolecular interactions and energetics. We expect that a large portion, perhaps the majority, of all poorly soluble drug molecules will require the consideration of mobility to allow for accurate predictions of relative stabilities in silico. Here, we demonstrated that this is feasible with readily available methodologies paving the way for molecular simulation to play a truly active role in the development, and finally the rational design, of ASD based drug formulations.

## Figures and Tables

**Table 1:** Details of the systems used here for MD simulations. Molecular content and average volumes for API–polymer blends, and pure systems.

Polymer	N${}_{\mathrm{pol}}$ ^*a*^	N${}_{\mathrm{mon}}$ ^*b*^	API	N${}_{\mathrm{api}}$ ^*c*^	m ^*d*^	w(API) ^*e*^	V ^*f*^
EEC	14	40	FLA	95	108,631	24.6	161.0
PAA	28	40	FLA	95	107,486	24.9	133.8
PSA	12	40	FLA	95	99,774	23.2	139.2
PVP	18	40	FLA	95	106,778	25.0	152.0
EEC	14	40	FLA	190	135,348	39.5	193.5
PAA	28	40	FLA	190	134,203	39.8	166.5
PSA	12	40	FLA	190	126,491	37.7	171.4
PVP	18	40	FLA	190	133,495	40.0	184.1
EEC	14	40	PAC	149	108,617	24.6	167.5
PAA	28	40	PAC	149	107,472	24.8	140.2
PSA	12	40	PAC	149	99,761	23.2	144.9
PVP	18	40	PAC	149	106,764	25.0	157.0
EEC	14	40	PAC	298	135,321	39.5	206.3
PAA	28	40	PAC	298	134,176	39.8	179.5
PSA	12	40	PAC	298	126,464	37.6	183.6
PVP	18	40	PAC	298	133,468	40.0	195.7
EEC	14	40	–	–	81,913	0	128.7
PAA	28	40	–	–	80,769	0	100.2
PSA	12	40	–	–	88,446	0	107.3
PVP	18	40	–	–	80,061	0	120.1
–	–	–	FLA	302	84,933	100.0	104.9
–	–	–	PAC	475	85,130	100.0	128.0

^*a*^ Number of polymer chains; ^*b*^ Number of monomers per polymer chain; ^*c*^ Number of API molecules; ^*d*^ Total mass of the system in atomic mass units; ^*e*^ API concentration in weight percent; ^*f*^ Average volume in MD simulations in nm${}^{3}$.

**Table 2:** Thermodynamic and kinetic descriptors from MD simulations of eight API–polymer blends compared to experimental literature data.

API	Polymer	AI25 ^*a*^	AI40 ^*b*^	<AI> ^*c*^	ΔE${}_{\mathrm{Coul}}$^*d*^	D ^*e*^	RMSF ^*f*^
FLA	EEC	100	100	87	−698.7	1.20	0.082
FLA	PAA	25	13	13	4863.8	0.42	0.068
FLA	PSA	0	0	15	1948.8	0.54	0.078
FLA	PVP	100	100	87	−3753.7	0.49	0.080
PAC	EEC	25	13	13	−704.0	3.73	0.0892
PAC	PAA	100	100	67	1945.4	1.51	0.0724
PAC	PSA	100	100	78	−1468.4	2.06	0.0790
PAC	PVP	100	94	49	−2590.1	1.25	0.0721

^*a*^ Amorphicity index at API concentration of 25 wt %; ^*b*^ Amorphicity index at API concentration of 40 wt %; ^*c*^ Average amorphicity index from six different API concentrations; ^*d*^ Calculated Coulomb contribution of the intermolecular mixing energy in kJ/mol; ^*e*^ Calculated API translational diffusion coefficient in 10${}^{-10}$ cm${}^{2}$/s; ^*f*^ Calculated average root mean square deviation of API in the MD trajectories after alignment of each API molecules’ center of mass in nanometer.

## Supplementary Materials

The following are available online at http://www.mdpi.com/1999-4923/10/3/101/s1, Figure S1: Literature data for eight different API molecules. Figures S2–S15: Convergence of various quantities calculated in MD simulations.

## Abbreviations

The following abbreviations are used in this manuscript:

API	Active Pharmaceutical Ingredient
ASD	Amorphous Solid Dispersions
PVP	Polyvinylpyrrolidone
HPMC	Hydroxypropyl Methylcellulose
DSC	Differential Scanning Calorimetry
MD	Molecular Dynamics
FH	Flory–Huggins
FLA	Flufenamic acid
PAC	Phenacetin
EEC	Eudragit E100
PAA	Polyacrylic acid
PSA	Poly (styrene sulfonic acid)
GAFF	General Amber Force Field
RESP	Restrained Electrostatic Potential
GROMACS	Groningen Machine for Chemical Simulations
LINCS	Linear Constraint Solver
PME	Particle Mesh Ewald
AI	Amorphocity Indices
VdW	Van der Waals
ΔE${}_{\mathrm{Coul}}$	change in Coloumb energy
ΔNHB	change in number of H-bonds
RMSF	average root mean square fluctuation
D	Diffusion coefficient
RT	Room Temperature
GPU	Graphic Processing Unit
