# Supplementary material for: Relative Contributions of Solubility and Mobility to the Stability of Amorphous Solid Dispersions of Poorly Soluble Drugs: A Molecular Dynamics Simulation Study
Source: Pharmaceutics. 2018 Jul 21;10(3):101. doi: 10.3390/pharmaceutics10030101 (PMC6161151; doi:10.3390/pharmaceutics10030101)
Supplement: Supplementary file 1 [file pharmaceutics-10-00101-s001.pdf]

pharmaceutics

MDPI

Supplementary material for "Relative Contributions of Solubility and Mobility to the Stability of Amorphous Solid Dispersions of Poorly Soluble Drugs: A Molecular Dynamics Simulation Study"

Michael Brunsteiner [1], Johannes Khinast [1,2] and Amrit Paudel [1,2,*]

1. Choice of Model Systems

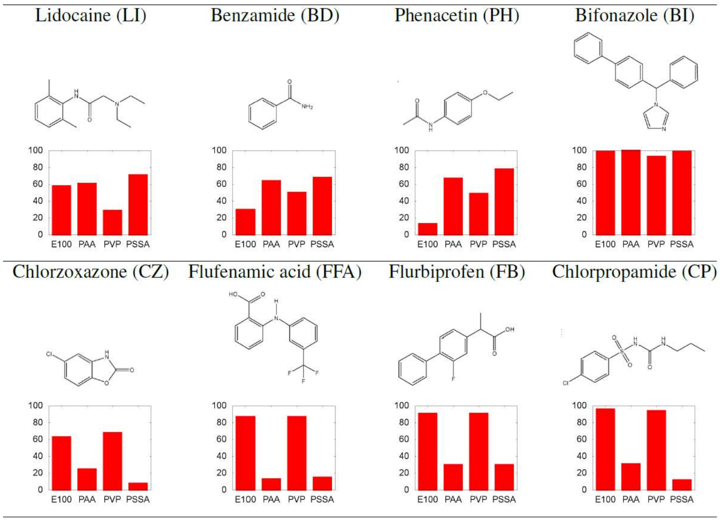

Figure S1. Literature data [51] for eight different API molecules, each formulated with four different polymer types. ASD stabilities are given as amorphicity index (AI) values that are each an average from measurements at several different API loadings.

2. Convergence

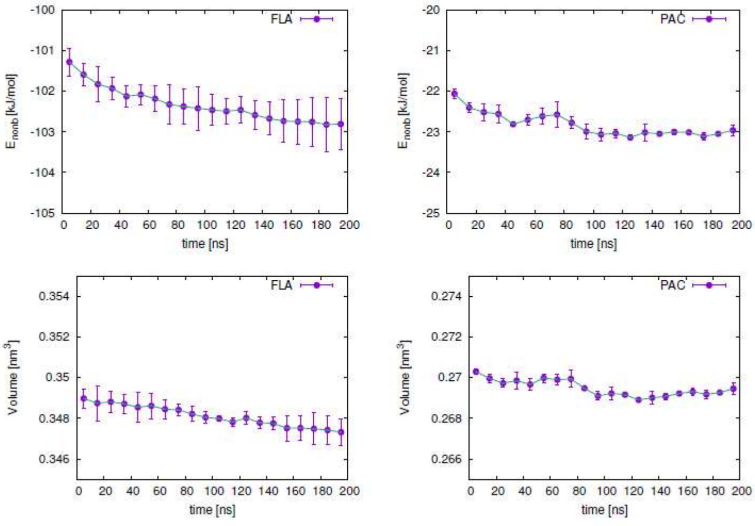

Figure S2. Convergence of simulations of pure APIs. Non-bonded energies (**top**) and volume per molecule (**bottom**).

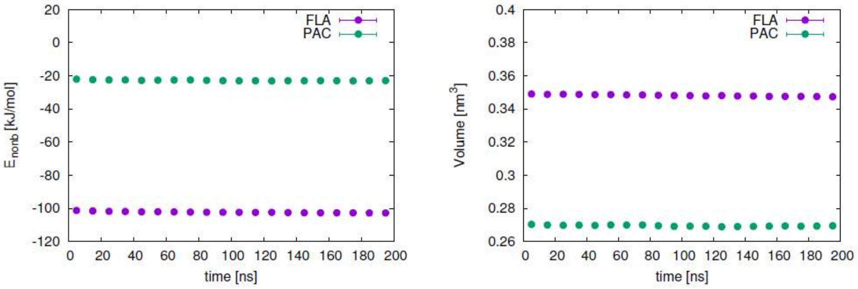

Figure S3. Convergence of simulations of pure APIs. Direct comparsion of data for the two APIs. Non-bonded energies (**left**) and volume per molecule (**right**).

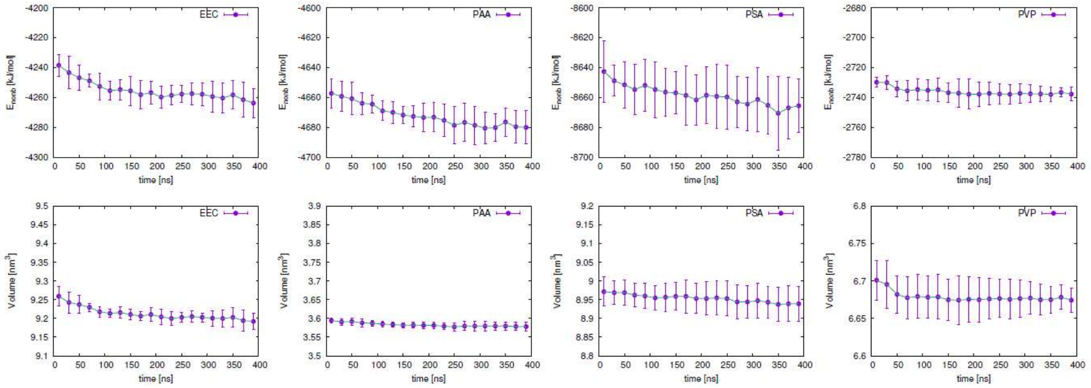

Figure S4. Convergence of simulations of pure polymers. Non-bonded energies (**top**) and volume per molecule (**bottom**).

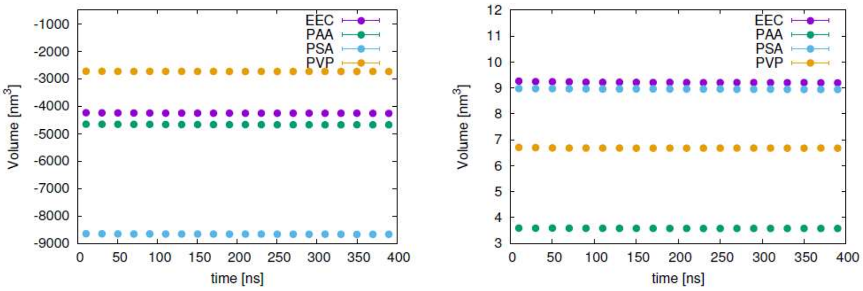

Figure S5. Convergence of simulations of pure polymers. Direct comparsion of data for the four polymer types. Non-bonded energies (**left**) and volume per molecule (**right**).

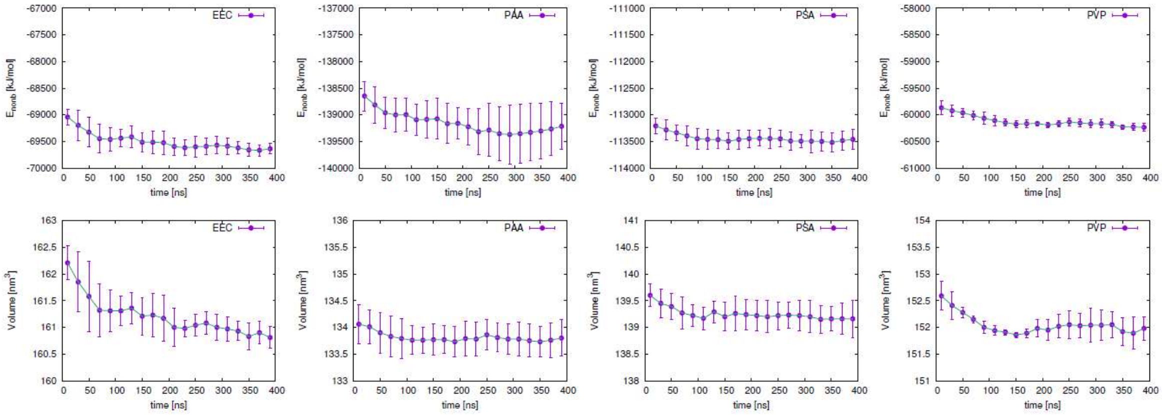

Figure S6. Convergence of simulations of blends of four polymer types with 25 w% FLA. Nonbonded energies (**top**) and volume per molecule (**bottom**).

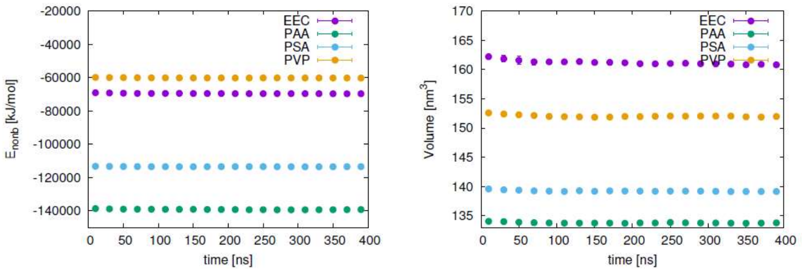

Figure S7. Convergence of simulations of blends of four polymer types with 25 w% FLA. Direct comparsion of data for the four polymer types. Non-bonded energies (**left**) and volume per molecule (**right**).

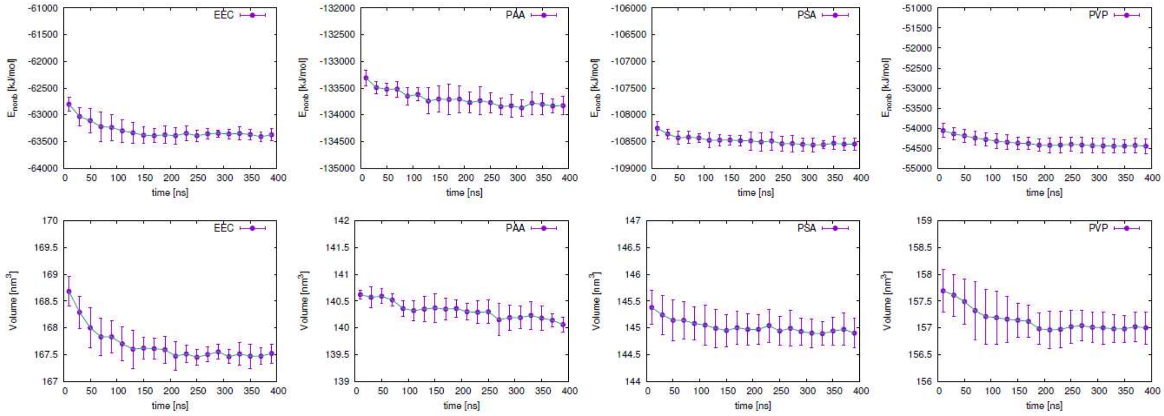

Figure S8. Convergence of simulations of blends of four polymer types with 25 w% PAC. Nonbonded energies (**top**) and volume per molecule (**bottom**).

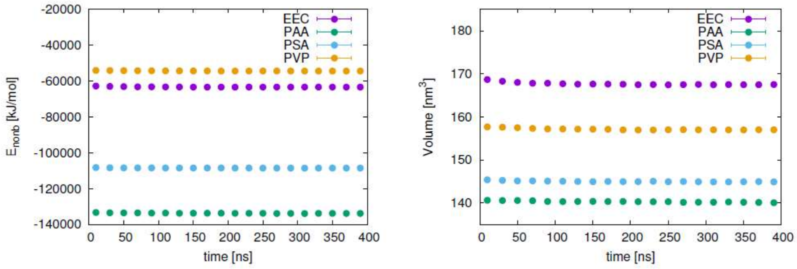

Figure S9. Convergence of simulations of blends of four polymer types with 25 w% PAC. Direct comparsion of data for the four polymer types. Non-bonded energies (**left**) and volume per molecule (**right**).

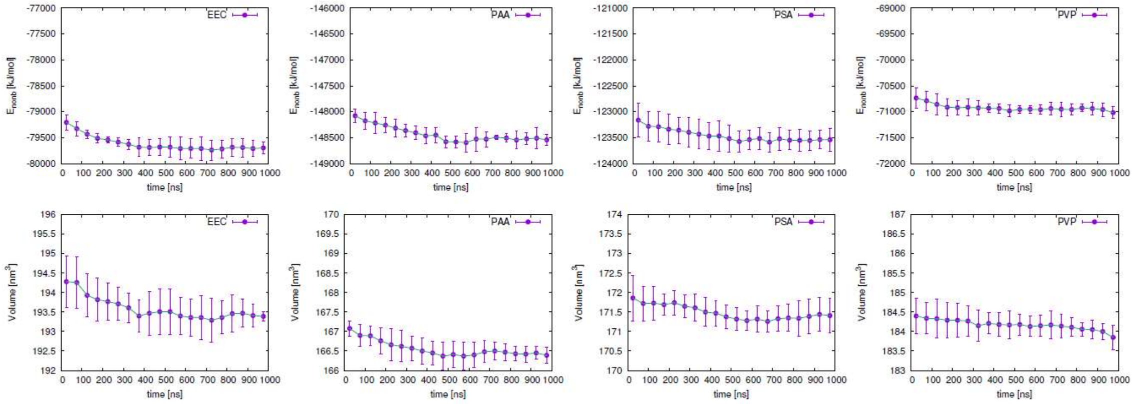

Figure S10. Convergence of simulations of blends of four polymer types with 40 w% FLA. Nonbonded energies (**top**) and volume per molecule (**bottom**).

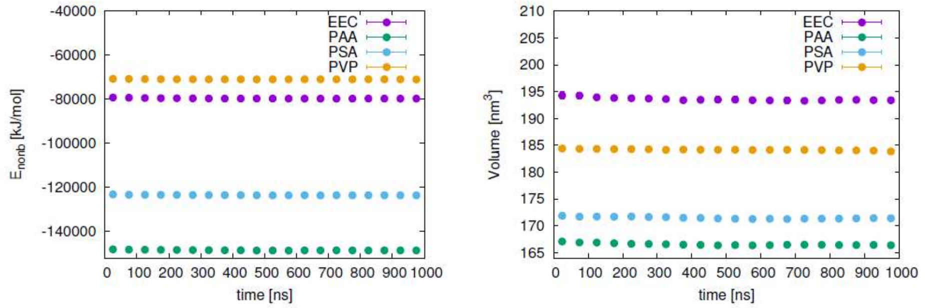

Figure S11. Convergence of simulations of blends of four polymer types with 40 w% FLA. Direct comparsion of data for the four polymer types. Non-bonded energies (**left**) and volume per molecule (**right**).

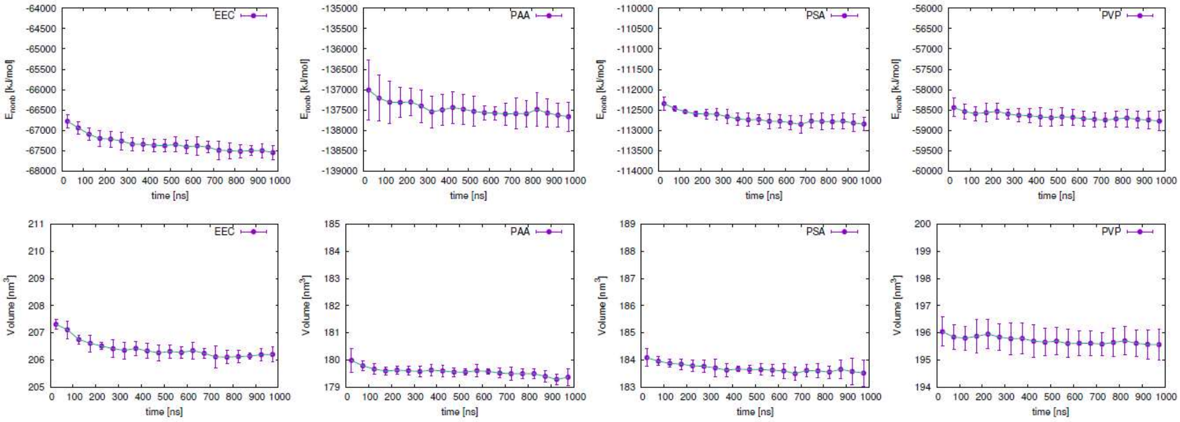

Figure S12. Convergence of simulations of blends of four polymer types with 40 w% PAC. Nonbonded energies (**top**) and volume per molecule (**bottom**).

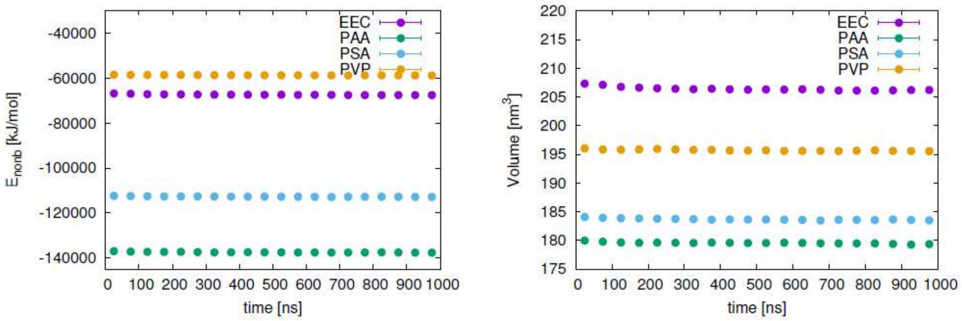

Figure S13. Convergence of simulations of blends of four polymer types with 40 w% PAC. Direct comparsion of data for the four polymer types. Non-bonded energies (**left**) and volume per molecule (**right**).

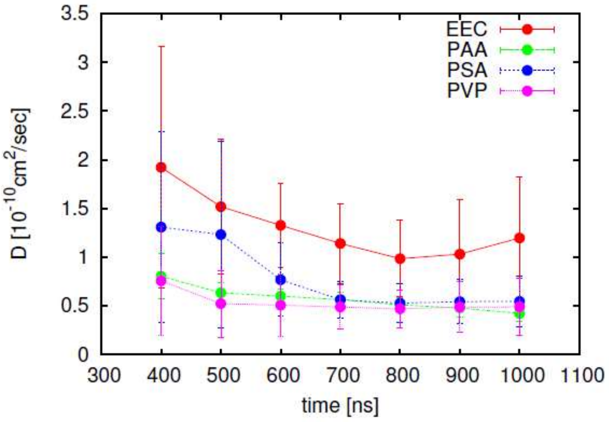

Figure S14. Convergence of API mobility calculations in blends of four polymer types with 40 w% PAC.

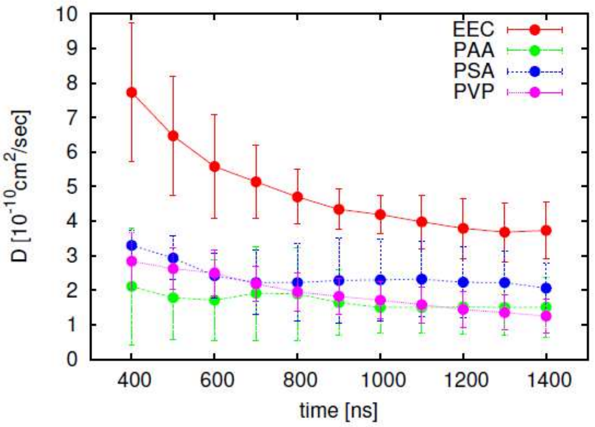

Figure S15. Convergence of API mobility calculations in blends of four polymer types with 40 w% PAC.

References

51. Eerdenbrugh, B.V.; Taylor, L. Small Scale Screening to Determine the Ability of Different Polymers to Inhibit Drug Crystallization upon Rapid Solvent Evaporation.Mol. Pharm. **2010**, 7, 1328–1337, doi:10.1021/mp1001153.
